# Application of ArcGIS 3D modeling technology in the study of land use policy decision making in China

**DOI:** 10.1038/s41598-023-47171-z

**Published:** 2023-11-24

**Authors:** Pengfei Cong, Dongming Zhang, Mingxuan Yi

**Affiliations:** Langfang Comprehensive Survey Center of Natural Resources, China Geological Survey, Langfang, 065000 China

**Keywords:** Climate sciences, Ecology, Environmental sciences, Natural hazards

## Abstract

In this paper, a land use management information system based on ArcGIS 3D modeling technology is constructed to process land use policy decisions through ArcSDE spatial data engine and Oracle relational database to realize a land use planning management information system. Using genetic algorithm in order to use for regional land use optimization allocation, the introduction of multi-intelligent body system in this algorithm will be able to enhance the optimization search ability of the algorithm and make the genetic algorithm to obtain land use planning supported. The behavior of the main body of the integrated land use planning decision maker will guide the development of the quantitative structure of land use in terms of spatial layout toward sustainability. The experimental results prove that the target is better than the other three types of scenarios under the integrated benefit model, then it is reduced by 18.67%, 15.98% and 16.61%, and the number of spatially contiguous areas is increased by 9.4%, 13.8% and 0.8%, respectively. The proposed model can reasonably configure the regional land use quantitative results and spatial layout, and coordinate the needs of different land use decision makers.

## Introduction

With the rapid progress of industrialization and urbanization, the utilization and conservation of land resources face multiple challenges, including the need for development and preservation, the imbalance between supply and demand, and the pressure to balance resource utilization with ecological protection^1–5^. Therefore, achieving optimal land use allocation is crucial for both informed decision-making and promoting sustainable land resource utilization and efficiency^6–9^. Optimal land use allocation is a complex spatial optimization problem that involves determining the allocation of land resources among different sectors and their spatial arrangement, considering the natural characteristics of the land and regional socioeconomic conditions^10–13^. Existing methods for optimal land use allocation can be classified into bottom-up and top-down models. Bottom-up models focus on simulating the decision-making processes of various land use entities and local-scale land use changes, employing approaches such as meta-automata and multi-intelligent body systems^14–18^. Top-down models focus on the overall regional situation and are able to consider the regional global objectives to obtain a series of optimal solutions. These models mainly include mathematical models such as linear programming and objective programming and intelligent algorithms such as genetic algorithms and particle swarm algorithms, which are characterized by large openness, high efficiency, and strong problem optimization solving ability^19–21^. According to previous studies^22^, the demand for 3D digital technology is increasing alongside the advancements in digital earth and digital city development. 3D GIS has emerged as a prominent trend due to its ability to provide a more realistic representation of the objective world compared to traditional 2D GIS^23^. It employs 3D modeling technology to present geospatial phenomena in a realistic manner, capturing both planar updates and vertical relationships between spatial objects. Additionally, 3D GIS offers unique functionalities for spatial browsing and analysis^24^. The core component of 3D GIS is the 3D spatial database, which requires careful consideration of factors such as aesthetics, shape, vegetation, building characteristics, and water resources during data design. Integration between 2 and 3D data dimensions, accurate positioning of 3D models, and the correspondence of spatial information are also important considerations^25^. Establishing a scientific, standardized, and comprehensive land use planning management information system using advanced technologies is crucial to meet the operational requirements of land use policy decision-making and overcome limitations of previous systems. This endeavor holds significant present and practical significance.

This article constructs an application based on ArcGIS 3D modeling technology for land use policy decision making research through a GIS software development platform combined with a land use planning management information system. In the process of platform construction, firstly, ArcSDE spatial data engine and Oracle relational database are used to process land use policy decision in order to realize land use planning management information system. Secondly, genetic algorithm is proposed to be used for optimal regional land use allocation and data analysis is performed based on the experimental results. Finally, the introduction of multi-intelligent body system in the genetic algorithm will be able to enhance the optimization search ability of the algorithm and make the genetic algorithm obtain the support of land use planning knowledge, synthesize the behavior of land use planning decision makers and guide the development of land use quantity structure in spatial layout to sustainable aspects. The paper introduces a new approach to land use management using ArcGIS 3D modeling, ArcSDE spatial data engine, and Oracle database. It incorporates genetic algorithms and multi-intelligent body systems to optimize regional land use allocation and considers land use planning knowledge. The model focuses on achieving sustainable land use by guiding the development of land use quantity structure in spatial layout. This research provides valuable insights for decision makers aiming to achieve optimal land use allocation.

This study aims to address the existing knowledge gaps and research limitations by developing a comprehensive land use planning management information system that integrates ArcGIS 3D modeling, genetic algorithms, and multi-intelligent body systems. The authors identified a lack of studies combining these elements to optimize regional land use allocation and guide decision-making towards sustainable land use. The depth of the study encompasses the construction and implementation of the information system, utilization of advanced technologies and algorithms, analysis of experimental results, and consideration of various factors in land use planning. The authors provide a thorough examination of the proposed model and its implications for decision makers.

## ArcGIS 3D modeling

### System structure

**Figure 1:**
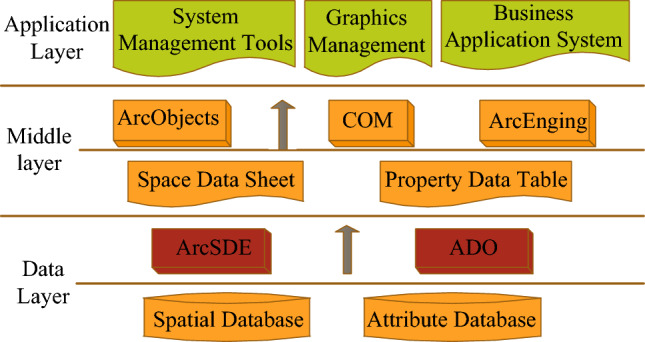
System architecture diagram.

ArcGIS, developed by ERSI, is a powerful platform with a robust and collaborative user community, offering extensive customization options for advanced developers. It supports various programming languages for customizing and extending its functionality, including the industry-standard VBA for script programming and customization. The ArcGIS geodatabase model, based on the ArcSDE application server, serves as a standard relational database for data management, acting as a bridge between ArcGIS and the relational database. This enables users to manage geographic information across multiple data management systems and makes the data accessible to all ArcGIS applications. In the land use information system, the GIS software development platform selected is ArcEngine of ArcGIS, the spatial data engine is ArcSDE, and the relational database chosen is Oracle, facilitating the creation of graphical and attribute databases. The system follows a Client/Server (C/S) architecture, logically divided into three layers: the application layer, middle layer, and database layer, as depicted in Fig. 1.

The specific analysis of Fig. 1 is as follows:

Based on ArcGIS for architecture, the application layer realizes GIS functions and regular business functions required for land update survey business, such as retrieval, query, browsing, etc. The application layer only communicates with the middle layer.

The land use planning management information system is structured into three layers: the data layer, the processing layer, and the presentation layer. The data layer utilizes ArcSDE spatial data engine and an Oracle relational database to store and manage land use data, policy decisions, and related information. The processing layer integrates ArcGIS 3D modeling technology and genetic algorithms to develop optimization models for regional land use allocation. It also incorporates multi-intelligent body systems to enhance the search capabilities of the genetic algorithm and incorporate land use planning knowledge. The presentation layer offers a user interface through a GIS software development platform, enabling decision makers to interact with the system and access relevant information. It may utilize 3D GIS technology to provide realistic representations of spatial layout and land use scenarios. The middle layer serves as a bridge between the application layer and the database layer. It facilitates information transfer and processing between the client and server, handling tasks such as extraction, transmission, and pre-processing of attribute and graphic data for the application layer. ArcSDE for Oracle9i acts as the channel for exchanging spatial data between the client program and the spatial database server. It provides a convenient means of organizing vector data into the database, establishing data links, and performing pre-processing of spatial data. The database layer is built on the Oracle9i large relational database system and consists of two components: the spatial database and the spatial data engine. The spatial database is used by Oracle to store various types of spatial data, while ArcSDE provides database services to efficiently operate both spatial and non-spatial data.

### Construction of 3D geodatabase

#### 1ArcSDE spatial database engine

ArcSDE is a tool that offers multi-user spatial data access for Oracle systems in land use planning management information systems. It enables the organization of the relational database model within the Oracle application, which can be accessed through a B/S (Browser/Server) or C/S (Client/Server) architecture. From a spatial data management perspective, ArcSDE supports a continuous spatial data model, allowing Oracle to store and manage extensive spatial data, including vector data, raster data, and metadata. The spatial data engine of ArcSDE utilizes a client/server architecture to facilitate communication between data. By integrating spatial data with attribute data, ArcSDE extends the relational data processing capabilities of Oracle, enabling unified management of both types of data.

#### Oracle relational database

**Figure 2:**
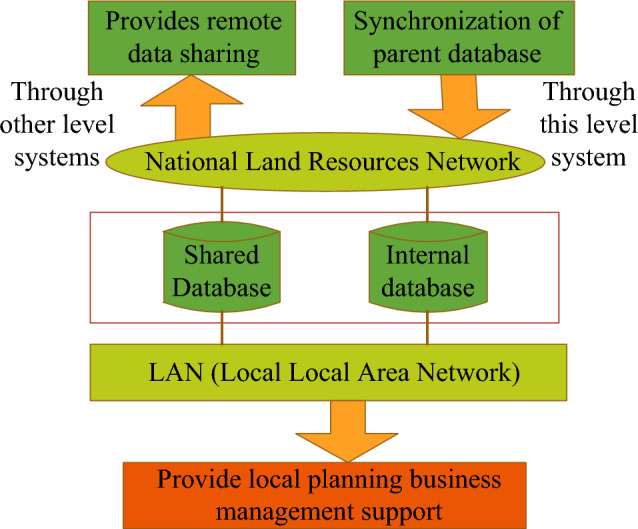
Oracles relational database.

The land use planning management database consists of an internal database and a shared database. It utilizes the Oracle relational database management system to handle data management tasks and performs relevant database operations using the Oracle SQL language. The database is shared with higher-level databases through a dedicated land resource network or with remote data through other systems at different levels. The relational database leverages its capabilities in managing massive data, transaction processing, record locking, concurrency control, and data warehousing to facilitate the integration of spatial and non-spatial data. This integration enables the realization of a real B/S (Browser/Server) or C/S (Client/Server) structure, ensuring connectivity and sharing across the entire land planning management database. Figure 2 illustrates the Oracle relational database diagram.

As can be seen from Fig. 2, the land use planning management database can be divided into system database, basic database, result database and auxiliary database by type.

1. The system library contains some operation parameters, codes, etc. necessary for system operation.
2. The basic database contains information such as land classification, location, tenure of the current land use data and extended information related to the project. In order to carry out information processing and extraction, thus realizing standardized storage of land use planning information data and information resource sharing.
3. The result database includes various land planning drawing result data and text result data. The drawing results mainly refer to the land planning use zoning, the location of planned transportation and water conservancy projects, the current status map of land use and other drawings.
4. The auxiliary database refers to the auxiliary database for normal operation and functioning, mainly graphical data, such as basic topographic data, land use status data, construction land approval data, etc., which can be directly called or called after conversion.

Genetic algorithm was used to conduct experiments on the land use of urban area B in province A between 2012 and 2021, and the experimental data included basic geographic information data such as slope and soil type. A 100 m*100 m raster was used to rasterize the study area and Markov chains were used to determine the transition probabilities between land use types in the study area, as shown in Table 1.

**Table 1:** Probability of conversion between regional land use types.

Present situation Land type	Optimized land type						
	Cultivated land	Garden plot	Woodland	Other agricultural land	Land used for building	Waters	Unused land
Cultivated land	–	0.3	0.5	0.5	0..6	0	0
Garden plot	0.4	0.1	0.3	0.1	0.4	0	0
Woodland	0	0.1	-	0.4	0	0	0
Other agricultural land	0.6	0	0	-	0	0	0
Land used for building	0	0	0	0	-	0	0
Waters	0	0	0	0	0	-	0
Unused land	0.8	0.1	0.2	0.2	0.8	0	-

## Land use policy decision algorithm

**Figure 3:**
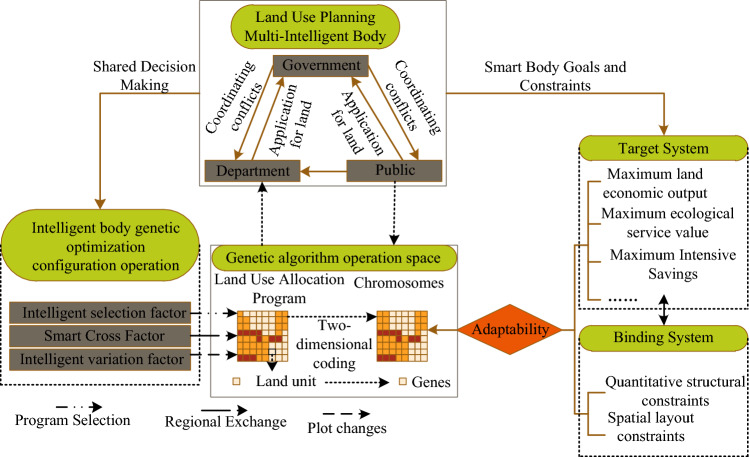
Multi-intelligence genetic algorithm frameworks.

The multi-intelligent body system is used to simulate the decision-making subjects involved in land use planning, and the genetic algorithm calculates the optimal regional land use allocation scheme with the assistance of multi-intelligent bodies. The genetic algorithm uses two-dimensional coding to characterize the geographic raster space, chromosomes correspond to land use allocation schemes, and genes characterize land units. The genetic algorithm provides a geospatial framework for multi-intelligent system decision making. The spatial layout of land use characterized by chromosomes and the natural, social and economic elements above constitute the geographic environment layer, which are observed and perceived by the land use planning multi-intelligent body. At the same time, the land use layout behavior of the multi-intelligence body in turn influences the chromosome, and a feedback relationship between the two forms a mutual operation, Fig. 3 shows the architecture of the multi-intelligence body genetic algorithm.

As can be seen from Fig. 3, the site layout planning comes to initialize the chromosomes, and their spatial decision-making behavior acts in the genetic optimal allocation operator of the intelligences to optimize the land use allocation scheme. The objectives and constraints of multiple intelligences constitute the objective system and constraint system of the model, and are translated into the fitness function of the genetic algorithm to guide the generation of the optimal land use allocation scheme and maximize the economic, social and ecological benefits of regional land use.

### Land use planning decisions

Based on the hierarchy of regional land use optimization allocation, the main body of land use planning decision making is divided into three levels: government, department, and public. The government, as the macro policy-making body, needs to determine the strategic direction of land use, designate land use control indexes and implement spatial control measures. Departments organize functional land use zoning and carry out sectoral land use layout planning, and the public realizes land unit allocation according to their individual interest needs. The model sets up these three types of intelligences, which do not occupy geospatial units, but participate in the land use planning decision-making process.

#### Government

The government acquires departmental and public land requests and coordinates their conflicts, as in Eq. (1).

1 $$C_{t} (i,j) = U_{d} (i,j,t) + \alpha \cdot U_{d} (i,j,t) + p_{t}$$

In Eq. (1), $C_{t} (i,j)$ denotes the utility function of cell $(i,j)$ on land class $t$ to the sector and the public. $\alpha$ denotes the degree of public participation $(0 \le \alpha \le 1)$, and a larger value indicates a higher degree of participation. $p_{t}$ is the sectoral priority $(p_{t} \ge 0)$ using land class $t$, and the higher the priority the more competitive the sector is.

As the number of applications from departments or the public for a site increases, its probability of using the site increases to reflect the government's full consideration of departmental requirements and public meanings in planning. The model simulates the process by adjusting the competition degree to achieve feedback operation among multiple intelligences as in Eq. (2).

2 $$C_{t}^{\prime } (i,j) = U_{d} (i,j,t) + n_{d} \cdot \Delta P_{d} + n_{p} \cdot \Delta P_{p}$$

In Eq. (2), $C_{t}^{\prime } (i,j)$ represents the adjusted competition degree. $n_{d}$ is the number of times the department applies for a site on the unit. $\Delta P_{d}$ is the size of the increase in competition per application. $n_{p}$ is the number of times the public applies for a site on the unit. $\Delta P_{p}$ is the size of the increase in competition per application.

#### Sector

Sectors pursue the maximization of their own sectoral interests, they apply for sites to the government, and coordinate with other sectors under the coordination of the government for the conflicts arising from site size and location choices. The dynamic random utility model and discrete choice model are used here to simulate the site layout behavior of the sector, and the selection probability of plot unit $(i,j)$, as in Eq. (3).

3 $$P(i,j,t) = \frac{{\exp (U_{d} (i,j,t))}}{{\sum {\exp (U_{d} (i^{\prime},j^{\prime},t))} }} = \frac{{\exp (R_{neigh} \cdot S_{ijt} )}}{{\sum {\exp (R_{neigh} \cdot S_{ijt} )} }}$$

In Eq. (3), $R_{neigh}$ is the proportion of the number of sectoral sites in the neighborhood to reflect the principle of spatial concentration and contiguity. $S_{ijt}$ is the suitability of the sectoral land use on the unit.

#### Public

The public can be mainly divided into two categories, urban residents and rural residents, with urban residents mainly participating in urban construction land layout decisions and rural residents mainly participating in rural land preparation decisions. The layout behavior of the public is basically the same as the sector, which is simulated using dynamic random utility model and discrete choice model, but it does not consider the neighborhood factor, and the selection probability of plot unit $(i,j)$, as in Eq. (4).

4 $$P(i,j,t) = \frac{{\exp (U_{p} (i,j,t))}}{{\sum {\exp (U_{p} (i^{\prime},j^{\prime},t))} }} = \frac{{\exp (R_{neigh} \cdot S_{ijt} )}}{{\sum {\exp (R_{neigh} \cdot S_{ijt} )} }}$$

### Optimize the configuration of the target system

The model is designed with 3 types of objective functions to maximize the economic, social and ecological benefits of regional land use.

1. Economic benefit is selected to maximize the total economic output of regional land use as the economic benefit objective, as in Eq. (5).
  5 $$F_{economy} = \sum {G_{t} } \cdot S_{t}$$
  In Eq. (5), $F_{economy}$ is the total economic output of regional land use/billion RMB. $G_{t}$ is the GDP per unit area of $t$ land use category, billion km^2^, which is obtained by various methods such as gray forecast, regression forecast and trend forecast. $S_{t}$ represents the area of regional $t$ land use category, km^2^.
2. Social benefits choose maximizing the degree of regional land intensification and saving as the social benefit objective, and use the neighborhood homogeneity index as the index, as in Eq. (6).
  6 $$F_{society} = \sum\limits_{i - 1}^{I} {\sum\limits_{j - 1}^{J} {A_{ij} } }$$
  In Eq. (6), $F_{society}$ is the target value of the regional land intensive saving degree. $I$ and $J$ are the scope of regional regular grid. $A_{ij}$ represents the number of parcels with the same land type in the neighborhood of parcel unit $(i,j)$. In the paper, the degree of intensive saving of construction land is mainly evaluated.
3. Ecological benefit is selected to maximize the regional organic carbon storage as the ecological benefit target, as in Eq. (7).
  7 $$F_{eco\log y} = \sum {C_{t} } \cdot S_{t}$$
  In Eq. (7), $F_{eco\log y}$ is the regional organic carbon storage volume/million tons. $C_{t}$ indicates $t$ land types per unit area to kill that organic carbon fixation, million tons km^2^.

### Optimize the configuration of the constraint system

The model constraints mainly include 3 types of constraints: quantity structure constraints, land type constraints and land class conversion rules.

1. The area of each land use type in the region should meet the land use quantity structure constraints, such as setting a red line for the amount of arable land to ensure food security and setting an upper limit for construction land to prevent excessive growth, as in Eq. (8).
  8 $$d_{k} (A) = D^{\prime}_{k} ,\sum {_{M} d_{k} (A) = D}_{0}$$
  In Eq. (8), $d_{k} (A)$ represents the number of grids with land type $k$ in the region, and $D^{\prime}_{k}$ represents the number of grids with land type $k$ in the optimized structure.
2. The value domain of the land type corresponding to the land type constraint grid, as in Eq. (9).
  9 $$S_{M} \in \left\{ {1,2, \ldots ,M} \right\}$$
3. Set constraints in areas such as basic farmland protection zones and ecological protection zones of rivers and lakes to prohibit unsuitable land use conversions, as in Eq. (10).
  10 $$0 \le p_{n} (m \to m^{\prime}) \le 1$$
  In Eq. (10), $p_{n} (m \to m^{\prime})$ represents the probability that the grid $S_{n}$ site type is converted from $m$ to $m^{\prime}$.

### Genetic optimization of intelligent body configuration

Intelligent body genetic optimization allocation operation, firstly, the department and the public observe and perceive the current land use allocation scheme, plan the layout according to the random utility model and discrete choice model, and submit the land use application to the government. The government determines the conflict area according to the land use application, coordinates the land use conflict based on the competition degree function, and updates the competition degree of land class. The model is designed with 3 categories of intelligent selection, intelligent crossover, and intelligent variation to achieve the optimization of the allocation scheme. The model evaluates the new generation of allocation schemes based on the land use optimization objective system and constraint system, and finally judges the termination conditions.

#### Intelligent selection operator

In the land use planning management system, an intelligent selection operator was developed to facilitate the selection of appropriate land use allocation options. This operator consists of two stages to effectively guide the evolutionary process. The first-stage selection operator employs the roulette wheel criterion. After the population of chromosomes undergoes crossover and mutation, the operator selects the chromosome with higher fitness as the parent for the next generation. The roulette wheel criterion assigns a probability value to each chromosome based on its fitness, and the selection process is akin to spinning a roulette wheel, where the likelihood of selection is proportional to the fitness value. By favoring chromosomes with higher fitness, this operator ensures that the most promising solutions have a higher chance of being selected for further evolution. The second-stage selection operator focuses on comparing chromosomes before and after crossover or mutation. After the completion of crossover or mutation, individual chromosomes are evaluated to determine their performance improvement or deterioration. This operator retains the chromosomes that show better performance after the evolutionary process and eliminates those with inferior performance. By doing so, it enhances the efficiency of the algorithm's search process by eliminating suboptimal or inefficient solutions. The combination of these two selection operators helps to guide the evolutionary process towards better land use allocation options. The first-stage operator ensures that chromosomes with higher fitness contribute to the next generation, while the second-stage operator further refines the population by eliminating weaker solutions. Together, they contribute to improving the overall efficiency and effectiveness of the algorithm in searching for optimal land use allocation solutions.

#### Intelligent crossover operators

**Figure 4:**
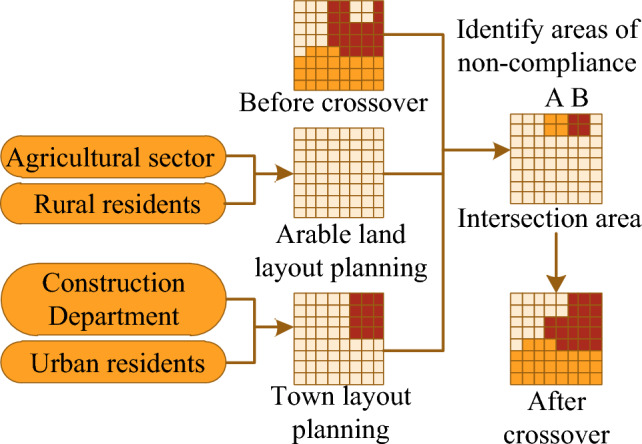
Intelligent cross operator.

The intelligent cross-calculus is mainly used to adjust the contradictions between the sectoral and public land use layout plans and the current land use allocation scheme, and Fig. 4 shows the intelligent cross-calculus diagram.

As can be seen from Fig. 4, in the departmental and public planning of arable land and town layout, there are some areas where the planned land types do not match with the current land use allocation scheme. The intelligent crossover operator screens a pair of 2 non-conforming areas with exactly opposite types and spatial proximity, such as the site types of A and B, for crossover to meet the planned needs.

#### Intelligent variational operators

**Figure 5:**
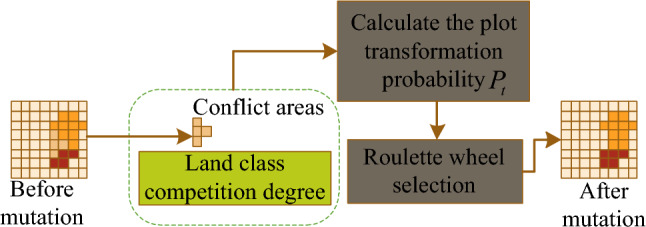
Intelligent variational operators.

The variation of land unit types is used to solve the conflict of different types of land layout. When departments and the public make land layout according to their respective needs, some parcels with good natural conditions and better location conditions may be selected by more than one, forming land conflict areas, Fig. 5 shows the intelligent variation operator diagram.

As can be seen from Fig. 5, the intelligent variation operator constructs the probability of parcels changing in different directions $p_{t} (i,j)$ based on the competitive degree function $C_{t} (i,j)$ set by the government for each land class, and uses a roulette wheel approach to resolve land conflicts, as in Eq. (11).

11 $$p_{t} (i,j) = \frac{{C_{t} (i,j)}}{{\sum {C_{t}^{\prime } (i,j)} }}$$

In order to achieve the action purpose, Agent (AI) $i$ will maximize its adaptation degree provided that the operating conditions of the algorithm are satisfied. In solving the urban land use space optimization allocation problem equation, the adaptation value $f(i)$ of Agent $i$ is, as in Eq. (12).

12 $$f(i) = eval(x) = z(x)p(x)$$

In Eq. (12), $z(x)$ is the weighted form of multiple objectives. $p(x)$ is the adaptive penalty function.

13 $$\left\{ \begin{gathered} z(x) = \sum\limits_{k = 1}^{q} {w_{k,i} f^{\prime}_{k,i} } (x) \hfill \\ p(x) = 1 - \frac{1}{m}\left[ {\frac{{\Delta b_{k,i} (x)}}{{\Delta b_{k,i}^{\max } }}} \right]^{\alpha } \hfill \\ \end{gathered} \right.$$

In Eq. (13), is the number of targets. $w_{k,i}$ is the weight of the $k$rd goal of individual $i$ in the group. $f^{\prime}_{k,i} (x)$ is the normalized form of the $k$th objective $f_{k,i} (x)$ of individual $i$ in the group. $\Delta b_{k,i} (x)$ is the violation value of the $k$th constraint for individual $i$ in the group. $\Delta b_{k,i}^{\max }$ is the maximum violation value of the $k$th constraint for individual $i$. $m$ is the number of constraints to be processed in the roundup. $\alpha$ can be considered as the index of the penalty function greater than 0, which can be set according to the actual situation and is generally taken as 1.

Each solution is considered as a particle of a certain volume in a $d$-dimensional search space. The current position of Agent $L_{i,j}$ in the solution space is the knowledge it possesses, and its position vector in the solution space of the optimal land use space allocation problem is denoted as $L_{i,j} = (L_{i,j + 1} ,L_{i,j + 2} , \ldots ,L_{i,j + d} )$. $L_{i,j + 1} ,L_{i,j + 2} , \ldots ,L_{i,j + d}$ represents the attribute values corresponding to the land use decision variables selected by Agent $L_{i,j}$. The flight speed of Agent $L_{i,j}$ is denoted as $V_{i,j} = (V_{i,j + 1} ,V_{i,j + 2} , \ldots ,V_{i,j + d} )$. $M_{i,j}$ is the Agent $L_{i,j}$ is the Agent with the maximum fitness in the neighborhood environment and $M_{i,j} = (m_{1} ,m_{2} , \ldots ,m_{n} )$. $P_{c}$ is the neighborhood cooperation probability. $P_{m}$ is the neighborhood competition probability $U(0,1)$ is a uniformly distributed random number, and if $U(0,1) < P_{m}$, the neighborhood competition operator is executed. If $U(0,1) < P_{c}$, the neighborhood cooperation operator is executed.

If $L_{i,j}$ satisfies Eq. (14), its position in the solution space remains unchanged, and conversely the position of $L_{i,j}$ in the solution space will be changed according to Eq. (15), and the type of Agent and the land use type of the grid it is on will be adjusted to be consistent with $M_{i,j}$.

14 $$f(L_{i,j} ) \ge f(M_{i,j} )$$

15 $$l_{k} = m_{k} + rand( - 1,1) \cdot (m_{k} - l_{k} ),k = 1, \ldots ,n$$

In Eqs. (14) and (15), $rand( - 1,1)$ is a random number between $( - 1,1)$. If $l_{k} < x_{k\min }$, then $l_{k} = x_{k\min }$. If $l_{k} > x_{k\min }$, then $l_{k} = x_{k\min }$. $x_{\min } = (x_{1\min } ,x_{2\min } , \ldots ,x_{n\min } )$ is the lower bound of the feasible solution space of the optimization problem, and $x_{\max } = (x_{1\max } ,x_{2\max } , \ldots ,x_{max} )$ is its upper bound. $L_{i,j}$ not only retains its original useful information, but also the industry fully absorbs the useful information of its neighbor $M_{i,j}$ to further increase its fitness value.

A hybrid crossover strategy is used to randomly select an intersection between $M_{i,j}$ and $L_{i,j}$, and the second half of the intersection is exchanged with each other while crossing each other at the intersection to obtain a new Agent. assuming that $M_{i,j}$ and $L_{i,j}$ are crossed at position $k$, the two resulting child Agents are divided into as, as in Eq. (16).

16 $$\left\{ \begin{gathered} M^{\prime}_{i,j} (m_{1} ,m_{2} , \ldots ,m^{\prime}_{k} ,l_{k + 1} ,l_{k + 2} , \ldots ,l_{n} ) \hfill \\ L^{\prime}_{i,j} = (l_{1} ,l_{2} , \ldots ,l^{\prime}_{k} ,m_{k + 1} ,m_{k + 2} , \ldots ,m_{n} ) \hfill \\ \end{gathered} \right.$$

In Eq. (16), $m^{\prime}_{k} = m_{k} + \beta (l_{k} - m_{k} )$, $l^{\prime}_{k} = v_{k} + \beta (u_{k} - v_{k} )$. $u_{k}$ and $v_{k}$ are the range of values of $l_{k}$, and $\beta$ is a random value within [0, 1].

17 $$V_{i,j,d}^{k + 1} = wV_{i,j,d}^{k + 1} + \varphi_{1} rand()(P_{i,j,d} - L_{{_{i,j,d} }}^{k} ) + \varphi_{2} rand()(G_{i,j,d} - L_{{_{i,j,d} }}^{k} )$$

18 $$L_{{_{i,j,d} }}^{k + 1} = L_{{_{i,j,d} }}^{k} + V_{i,j,d}^{k}$$

Equations (17) and (18) in, the subscript $k$ indicates the number of iterations. $w$ is the inertia constant. $\varphi_{1}$ and $\varphi_{2}$ are the learning factors, which regulate the maximum step of flight in the direction of the individual pole and the global pole, respectively. $rand()$ is a random number between (0,1). $L_{{_{i,j,d} }}^{k + 1}$ and $V_{i,j,d}^{k + 1}$ are the $d$-dimensional components of the current position and velocity of the Agent in the $k$ th iteration, respectively. $P_{i,j,d}$ The $d$-dimensional component of the optimal current position found by the Agent itself. $G_{i,j,d}$-dimensional optimal Agent in the population.

The $d$-dimensional component of the current position. By exchanging information with the global optimal Agent, it will speed up the information transfer of the Agent in the whole environment and improve the convergence of its algorithm.

## Experimental design and analysis of results

### Land use conversion probability

Genetic algorithm was used to conduct experiments on the land use of urban area B in province A between 2012 and 2021, and the experimental data included basic geographic information data such as slope and soil type. A 100m*100m raster was used to rasterize the study area and Markov chains were used to determine the transition probabilities between land use types in the study area, as shown in Table 1.

As can be seen from Table 1, with the support of ArcGIS technology, a land use management information system software, the hierarchical analysis method is used to calculate the weights of different land classes under four scenarios of maximizing economic benefits, maximizing social benefits, maximizing ecological benefits and maximizing comprehensive benefits, which is used to determine the allocation order of land classes. The Analytic Hierarchy Process (AHP) was utilized to determine the relative weights of various land types across four scenarios: maximizing economic benefits, maximizing social benefits, maximizing ecological benefits, and maximizing comprehensive benefits. AHP is a decision-making technique that systematically compares and prioritizes multiple criteria. By employing AHP, the researchers assigned importance values to each land type based on its contribution to the specific scenario's objective. These importance values were then utilized to establish the prioritized order of land classes, presenting a structured and unbiased approach to land use decision making. The integration of AHP with ArcGIS technology and genetic algorithms synergistically improved the precision and effectiveness of the land use management information system, enabling well-informed and optimized land allocation decisions.

### Optimize land layout

The spatial distribution outcomes vary under different land use scenarios while maintaining equal optimization objective weights. When prioritizing economic benefits, surveillance land exhibits noticeable clustering, and new construction land is contiguous with existing construction land. In scenarios emphasizing social benefits, the proportion of arable land and construction land is significantly higher than other types, leading to intense spatial competition in the urban fringe area and reduced contiguity. Maximizing ecological benefits assigns higher weights to garden land and forest land, resulting in significantly higher spatial agglomeration and suitability compared to other land types. In the pursuit of comprehensive benefits, the clustering of arable land, construction land, and ecological land is relatively balanced, yet falls short of the optimal targets in the other three modes. Detailed optimal allocation results for different land use scenarios are presented in Table 2.

**Table 2:** Optimal configuration under different scenario models.

Scene	MCB	MSC	Weighted sum	Number of partitions	Convergence times
Economic performance	194,810	20,399	0.47	1266	473
Social results	191,546	21,074	0.52	1218	483
Ecological benefit	190,317	20,916	0.54	1374	447
Comprehensive benefits	209,840	25,081	0.38	1389	557

As can be seen from Table 2, the MCB (modular adaptability test version) objective in the integrated benefit model is better than the other three types of scenarios, then it is reduced by 18.67%, 15.98% and 16.61%, respectively, and the number of spatially contiguous areas increases by 9.4%, 13.8% and 0.8%. The combined benefits need to take into account the suitability and agglomeration of all land classes, which affects the genetic algorithm and convergence efficiency.

**Figure 6:**
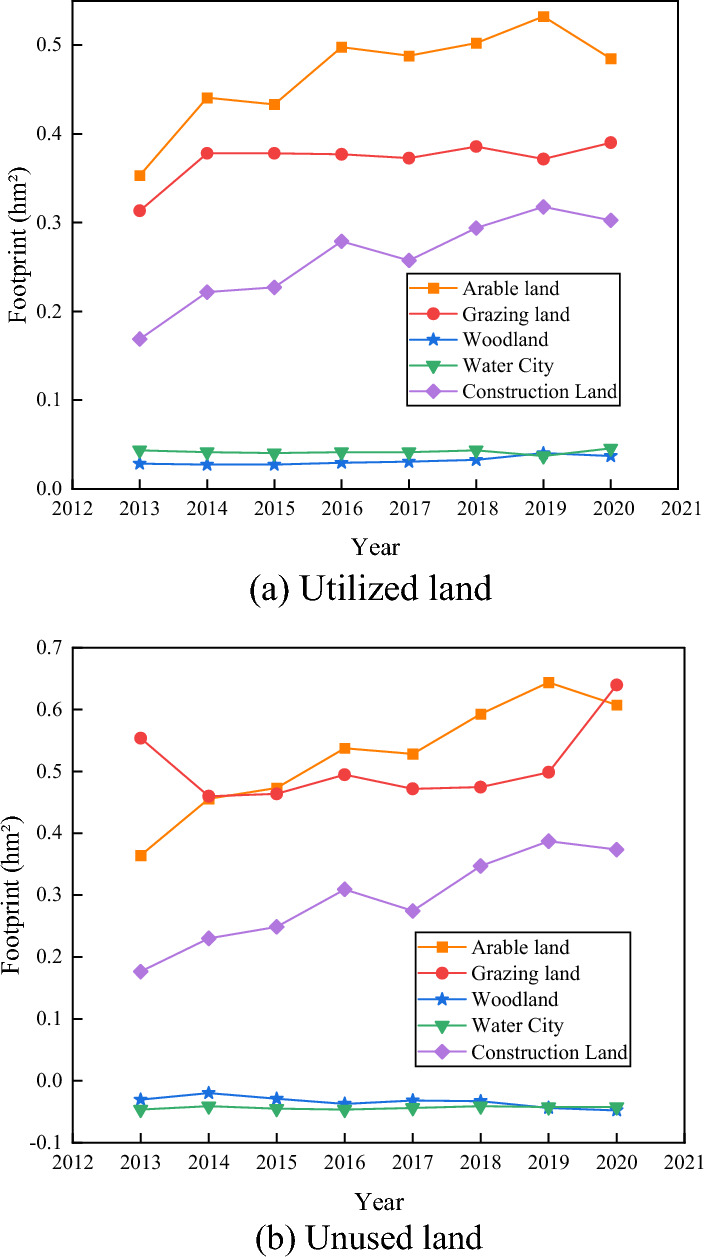
Land use optimization configuration layout.

Under the comprehensive benefit model, a comparative analysis of the optimized allocation scheme and the current spatial layout reveals that the spatial layout of unused land, arable land and construction land changed significantly during the optimization process. Among them, there is a slight increase in the quantity of arable land and a significant improvement in quality. Among the new arable land, the largest proportion comes from unused land and other agricultural land, indicating that the unused land in the study area has greater development potential, and Fig. 6 shows the layout of the optimal land use allocation.

As can be seen from Fig. 6a, the land use policy decision algorithm and the comparison chart show that arable land and pastureland are the main force of land use in the experimental area, and arable land, pastureland waters, and construction land all show a continuous growth trend year by year. Among them, pastureland is the fastest growing, increasing by 0.318 snhm^2^ from 2013 to 2020, with a growth rate of 148%. The rise of five types of land is pastureland > construction land > cultivated land > waters, and only forest land footprint shows a decreasing state.

As can be seen from Fig. 6b, the sources of unused land and other agricultural land account for a relatively large proportion of the new arable land. Although the arable land area decreased by 10,300,000 hm^2^ in the conversion of unused land types, the production of arable land products did not decrease significantly, which is directly related to the improvement of arable land output efficiency. Construction land newly increased to 16,000 hm^2^, but the construction land footprint, although slightly decreasing in 2013, 2014, and 2017 compared to the previous year, remained a continuous increase in general. In contrast, the forest land footprint shows a decreasing trend each year, decreasing by 0.05 snhm^2^ in 7 years. In the experimental area, arable and pastureland are the main land use types, with pastureland growing the fastest. Construction land and cultivated land also show growth, while forest land decreases. Unused and agricultural land contribute to new arable land, offsetting the decrease in arable area. Arable production remains stable, indicating improved efficiency. Construction land increases overall, with slight fluctuations. Forest land consistently decreases. The analysis highlights dominant land use types, growth rates, and observed changes in the experimental area.

Several works present recent advancements in a wide range of research areas, encompassing subjects such as unsupervised data modeling, track prediction methods, denoising techniques, imaging methods for space debris, discrimination between different types of ices^26–30^. Also, the underground space utilization, spectrum allocation pricing policies, task offloading strategies, crop mapping utilizing satellite imagery, echo detection systems for LiDAR, buffering algorithms, flight trajectory prediction, thermospheric density distribution has been investigated^31–35^. The urban heat islands, lake boundary prediction, water quality assessment, wafer manufacturing, source rock characterization, natural gas origins, and the coupled transfer of water, heat, and solute in saline loess^36–40^. The land use conversion rate and the optimized allocation results are overlaid and compared to analyze the allocation scheme and spatial layout. In general, the changed land use is mainly arable land, pasture land and construction land, while the change of water and forest land is not significant^41–45^. A wide range of topics have been explored in various research articles, including investigations on the mechanical properties of polymer membranes with nanoparticle reinforcement, scaffold fabrication using 3D printing and freeze-drying techniques, stability analysis of ceramic materials reinforced with nanoparticles, effects of graphene and copper oxide nanoparticles on composites, optimization of drone delivery systems, the relationship between knowledge management and operational performance in medical tourism, impacts of the COVID-19 pandemic, and catalyst regeneration using supercritical CO_2_^46–50^. Experimental results demonstrate that a land use optimization allocation model, based on a multi-intelligent genetic algorithm, effectively allocates the quantitative structure and spatial layout of regional land use. This approach significantly enhances the economic, social, and ecological benefits of land use while promoting sustainable utilization of land resources^51–55^. It ensures compliance with the red line requirements for retaining arable land and increases the quantity of high-quality farmland. The collaborative decision-making process involving different types of intelligence caters to the needs of government, departments, and the public at all levels^56, 57^.

## Conclusion

In this paper, we propose to establish a graphical database and an attribute database by establishing a land use planning management information system with ArcEngine of ArcGIS as the GIS software development platform, ArcSDE as the spatial data engine, and Oracle selected as the relational database of the land use information system. The application of land use policy decision making is studied and the design effect of the method is verified in the process of experiments. The conclusion of the experiment shows that the MCB target is better than the other three types of scenarios in the comprehensive benefit model, while the MSC is reduced by 18.67%, 15.98% and 16.61%, and the number of spatially contiguous areas is increased by 9.4%, 13.8% and 0.8%, respectively. The unused land within newly available arable areas demonstrates significant development potential, as its increased proportion indicates. This finding suggests that the land use optimization allocation model, employing a multi-intelligent body genetic algorithm, effectively distributes the regional land use quantity structure in spatial layout. Consequently, this model brings about noticeable enhancements in the economic, social, and ecological benefits of regional land use, thereby promoting the sustainable utilization of regional land resources.

## Data Availability

All data generated or analysed during this study are included in this published article.
